# Asynchronous responses of microbial CAZymes genes and the net CO_2_ exchange in alpine peatland following 5 years of continuous extreme drought events

**DOI:** 10.1038/s43705-022-00200-w

**Published:** 2022-11-16

**Authors:** Zhongqing Yan, Enze Kang, Kerou Zhang, Yanbin Hao, Xiaodong Wang, Yong Li, Meng Li, Haidong Wu, Xiaodong Zhang, Liang Yan, Wantong Zhang, Jie Li, Ao Yang, Yuechuan Niu, Xiaoming Kang

**Affiliations:** 1 Wetland Research Center, Institute of Ecological Conservation and Restoration, Chinese Academy of Forestry, 100091 Beijing, China; 2 Sichuan Zoige Wetland Ecosystem Research Station, Tibetan Autonomous Prefecture of Aba, Ngawa, 624500 China; 3 Beijing Key Laboratory of Wetland Services and Restoration, 100091 Beijing, China; 4 College of Life Sciences, University of Chinese Academy of Sciences, 100049 Beijing, China; 5 Information Center of Ministry of Ecology and Environment, 100029 Beijing, China; 6 Sino-Danish Centre for Education and Research, University of Chinese Academy of Sciences, 100049 Beijing, China; 7 State Key Laboratory of Mycology, Institute of Microbiology, Chinese Academy of Sciences, 100101 Beijing, China

**Keywords:** Biogeochemistry, Climate-change ecology, Microbial ecology

## Abstract

Peatlands act as an important sink of carbon dioxide (CO_2_). Yet, they are highly sensitive to climate change, especially to extreme drought. The changes in the net ecosystem CO_2_ exchange (NEE) under extreme drought events, and the driving function of microbial enzymatic genes involved in soil organic matter (SOM) decomposition, are still unclear. Herein we investigated the effects of extreme drought events in different periods of plant growth season at Zoige peatland on NEE and microbial enzymatic genes of SOM decomposition after 5 years. The results showed that the NEE of peatland decreased significantly by 48% and 26% on average (*n* = 12, *P* < 0.05) under the early and midterm extreme drought, respectively. The microbial enzymatic genes abundance of SOM decomposition showed the same decreasing trend under early and midterm extreme drought, but an increasing trend under late extreme drought. The microbial community that contributes to these degradation genes mainly derives from *Proteobacteria* and *Actinobacteria*. NEE was mainly affected by soil hydrothermal factors and gross primary productivity but weakly correlated with SOM enzymatic decomposition genes. Soil microbial respiration showed a positive correlation with microbial enzymatic genes involved in the decomposition of labile carbon (n = 18, *P* < 0.05). This study provided new insights into the responses of the microbial decomposition potential of SOM and ecosystem CO_2_ sink function to extreme drought events in the alpine peatland.

## Introduction

Global climate models predict an increase in the frequency of extreme drought events in the future [1]. Extreme drought seriously affects the pool, flux and process of the terrestrial carbon (C) cycle, especially can significantly reduce the intensity of terrestrial ecosystem C sinks and even convert them into C sources [2]. Peatlands are C-rich ecosystems that cover 185–423 million hectares of the earth’s surface [3, 4]. Although peatlands occupy only 3% of the land surface, they maintain huge amounts of 600–700 Gt C storage, which exceeds the C storage of global vegetation and is close to the carbon dioxide (CO_2_) storage in the atmosphere [5, 6]. Peatlands are very sensitive to extreme drought events. If oxygen (O_2_) is introduced through drought, the accompanying losses are CO_2_ and dissolved organic carbon (DOC) from peatlands [7].

Net ecosystem CO_2_ exchange (NEE) refers to the changes in C exchange between terrestrial ecosystems and atmospheric systems caused by the combination of plant photosynthesis, C storage in the canopy air, and C emissions derived from biological and non-biological respiration. Therefore, NEE characterizes the level of the terrestrial ecosystem’s ability to absorb atmospheric CO_2_. Furthermore, the seasonal timing of extreme drought events plays an essential role in affecting the structure and functioning of ecosystems, yet remains little explored [8, 9]. Previous studies have indicated that climate variability occurring in the early or midterm stage of the growing season could have larger impacts than climate variability late in the growing season [10, 11]. Despite such evidence regarding the importance of the seasonal timing of drought, our understanding of the changes in the NEE under extreme drought events at different times of the growing season is very limited.

Soil microbial communities are pivotal in mediating numerous essential ecosystem functions. High-throughput DNA sequencing provides an opportunity to characterize and quantify the potential functional properties of soil microorganisms [12] and ecosystem functions [13] deeply. Previous studies showed that drought stimulates microbial growth, causing the breakdown of soil organic matter (SOM) and the release of CO_2_ in a biogeochemical cascade [14, 15]. Carbohydrate active enzymes (CAZymes) are key enzymes in the degradation of SOM, the pool size of soil organic carbon (SOC) depends on the balance between the formation of SOM from the decomposition of plant litter and its mineralization to inorganic C by releasing CO_2_ to the atmosphere [16], which may furtherly influence the NEE. The family classification and annotation of CAZymes is now primarily used to analyze, understand, and compare the ability of organisms or communities to assemble and decompose complex carbohydrates. To facilitate studies of such CAZymes, these proteins have been grouped into classes and families based on sequence similarity in the CAZy database [17, 18]. Characterizing these enzyme groups associated with the conversion of carbohydrates in nature will improve our understanding of the significant roles of enzymatic decomposition potential of microorganisms and how they regulate the peatland’s C cycle [19, 20].

In this study, the in-situ experiment was carried out in Zoige peatland, which is the largest alpine peatland in the world due to the unique climatic, hydrological, geomorphic and soil conditions of the region [21]. We combined measurements of NEE and microbial CAZymes functional genes following 5-year of extreme drought events simulation. The objectives of this study are to (i) determine the variation of the NEE in alpine peatland under extreme drought manipulation; (ii) characterize the microbial CAZymes functional genes and taxa that participate in SOM decomposition; (iii) evaluate the contribution of soil microbial organic matter decomposition potential in regulating ecosystem CO_2_ sink function.

## Material and methods

### Study area and experimental design

The location of the peatland investigated herein is found at 3430 m above sea level, which is located in the Zoige plateau (33°47′56.61″N, 102°57′28.43″E). The mean annual temperature and mean annual precipitation are −1.7–3.3 °C and 650–750 mm, respectively, and about 90% of annual precipitation occurs from April to September. The state has a short growing season (July to September). The soil types are meadow soil and peat swamp soil, and the vegetation is dominated by *Kobresia* and *Gramineae*.

We have collected rainfall statistics for the past 50 years (China Meteorological Information Network). A daily rainfall of less than or equal to 3 mm was defined as non-effective rainfall, and the duration of non-effective rainfall was defined as 32 days (where days without effective rainfall represented drought duration). The experiment consisted of control with ambient precipitation, named E_CK, M_CK, and L_CK for the different periods of plant growth. The simulated extreme drought occurred from June 18 to July 20 (ED: early extreme drought), July 20 to August 23 (MD: midterm extreme drought), and August 23 to September 25 (LD: late extreme drought) in 2019, following 5 years of continuous extreme drought events from 2014 to 2018. Precipitation was sheltered by a transparent awning (length × width × height: 2.5 m × 2.5 m × 1.8 m). The transmittance of the shielding material is greater than 90%. Each treatment had three randomly selected duplicate plots (2 m × 2 m).

### CO_2_ fluxes measurement

NEE was measured between 10:00 and 15:00 from June 18 to September 25 in 2019, with approximately a one-week interval between each measurement. One square aluminum frame (0.5 m × 0.5 m) was inserted in the soil at 2–3 cm depth in each plot to provide a flat base between the soil surface and the CO_2_ sampling chamber. We measured NEE and ecosystem respiration (Re) with a cubic 0.125 m^3^ transparent chamber (0.5 m on each side) attached to a microportable greenhouse gas analyzer (GLA131, ABB, Canada) that covered all vegetation within the aluminum frames. The CO_2_ flux observed directly from the transparent static box represents the NEE, light shading static-chamber with an opaque cloth observed CO_2_ flux represents the Re. During the measurement, two small fans were used to mix the air inside the chamber, and CO_2_ concentration was recorded continuously for 120 seconds after reaching steady-state conditions. During the measurement interval, the air temperature in the test chamber increased by less than 0.2 °C.

The fluxes (*F*) of NEE and Re are calculated from the linear slope indicating the change of gas concentration over time as follows:

$$F = \frac{{dc}}{{dt}}\cdot \frac{M}{{V_0}}\cdot \frac{P}{{P_0}}\cdot \frac{{T_0}}{T}\cdot H$$

The unit of NEE and Re is mg m^−2^ h^−1^. *dc/dt* is the slope of the gas concentration over time (ppm h^−1^); *M* is the molar mass of the measured gas (g mol^−1^); *P* is the atmospheric pressure at the sampling point (Pa); *V*_*0*_, *P*_*0*_, and *T*_*0*_ represent the standard molar volume (22.41 L mol^−1^), standard atmospheric pressure (101,325 Pa), and absolute temperature under the standard atmospheric pressure, respectively; *T* is the absolute temperature inside the chamber, and *H* is the effective height of the chamber (m).

Gross primary productivity (GPP) is the difference between Re and NEE. Defining the NEE positive as net CO_2_ release from the ecosystem to the atmosphere and negative as net absorption, the GPP can be defined as:

$${{{{{{{\mathrm{GPP}}}}}}}} \, { = {{{{{{{\mathrm{Re}}}}}}}} - {{{{{{{\mathrm{NEE}}}}}}}}} $$

Total soil respiration (Rs) and microbial respiration (Rm) were measured by a soil greenhouse gas flux monitoring system PS-9000 (Beijing LICA United Technology Limited, China), which consisted of an infrared gas analyzer and a respiratory chamber (SC-11). Polyvinylchloride (PVC) collars 20 cm in diameter and 10 cm or 40 cm high were inserted into the soil at 2 cm depth near the aluminum frames in May 2018, which were used in Rs or Rm measurements, respectively. To eliminate the effects of plant respiration, the aboveground plant within the shallow collar was cut off and removed for Rs measurement every time. After one year, when the deep PVC tubes cut off old plant roots while preventing new roots from growing into the tubes, the CO_2_ efflux measured above these tubes represented Rm.

### Environmental variables measurement

Soil samples at 0–10 cm, 10–20 cm and 20–30 cm of every plot were gathered at the end of each extreme drought event for soil biochemical analyses. One part of the soil sample was air-dried for SOC and total nitrogen (TN) analyses. The other part was passed through a 2-mm sieve to determine DOC, nitrate (NO_3_^-^), ammonium (NH_4_^+^), and microbial biomass carbon (MBC). SOC and TN were determined by a soil C/N element analyzer (Vario EL III, Elementar, Germany) through dry combustion of samples (100-mesh). DOC was calculated for soil water extracts. A fresh sample of 15 g was placed in 150 ml of ultrapure water, oscillated at 15 °C for 24 h, screened using a prebaked 0.7-mm glass fiber filter (GF/F, Whatman, UK), and then analyzed by an elemental TOC analyzer (LiquiTOC II, Germany). Soil NH_4_^+^ and NO_3_^-^ concentrations were determined by an auto-analyzer (SEAL-AA3, Germany) from 2-mol L^−1^ KCl extracts. MBC was determined by an elemental TOC analyzer (LiquiTOC II, Germany), using the chloroform fumigation-extraction method, which was also introduced in our previous study [22, 23]. Soil temperature was measured at depths of 5, 10 and 20 cm using type-K thermocouples (Campbell Scientific, Logan, UT, USA). The soil volumetric water content (SWC) at depths of 5, 10 and 20 cm was assessed via a TDR 300 moisture meter (Spectrum, USA) on site.

### DNA extraction, library construction, and metagenomic sequencing

Three soil cores from the upper 10 cm were randomly sampled at the end of each extreme drought event from each plot, soil cores from the same plot were mixed, homogenized, sieved through a 2.0 mm mesh size sieve, and immediately stored at −20 °C prior to DNA extraction. Total genomic DNA was extracted from soil samples using the E.Z.N.A. Soil DNA Kit (Omega Bio-Tek, Norcross, GA, U.S.). The concentration and purity of extracted DNA were determined by TBS-380 and NanoDrop2000 respectively. The quality of the DNA extract was determined by 1% agarose gel.

DNA extracts were segmented to an average size of about 400 bp using Covaris M220 (Gene Company Limited, China) for the construction of paired terminal libraries. NEXTFLEX Rapid DNA-Seq was used to construct the paired-end library (Bioo Scientific, Austin, TX, USA). Paired-end sequencing was performed on Illumina NovaSeq 6000 (Illumina Inc., San Diego, CA, USA) at Majorbio Bio-Pharm Technology Co., Ltd. (Shanghai, China) using NovaSeq Reagent Kits (www.illumina.com).

### Sequence quality control and genome assembly

The paired-end Illumina reads were adjusted, and the sequences with low quality (length < 50 bp or mass value < 20 or containing N bases) were removed with fastp [24] (https://github.com/OpenGene/fastp, version 0.20.0). Metagenomics data were assembled using MEGAHIT [25] (https://github.com/voutcn/megahit, version 1.1.2), which makes use of succinct de Bruijn graphs. Contig lengths greater than or equal to 300 bp were selected as the final assembly result, and contig was then used for further gene prediction and annotation.

### Gene prediction, taxonomy, and functional annotation

MetaGene was used to predict Open reading frames (ORFs) from each assembled contig [26] (http://metagene.cb.k.u-tokyo.ac.jp/). The predicted ORFs with length ≥100 bp were retrieved by NCBI translation table and translated into amino acid sequence. (http://www.ncbi.nlm.nih.gov/Taxonomy/taxonomyhome.html/index.cgi?chapter=tgencodes#SG1).

A non-redundant gene catalog was constructed using CD-HIT [27] with 90% sequence identification and 90% coverage (http://www.bioinformatics.org/cd-hit/, version 4.6.1). Using SOAPaligner [28] (http://soap.genomics.org.cn/, version 2.21), post-quality control readings were mapped to a non-redundant gene catalog with 95% identity and gene abundance in each sample was assessed.

The representative sequences of the non-redundant gene catalog were compared with NCBI NR database, and the cutoff value of e value was 1e^−5^ for taxonomic annotations using DIAMOND [29] (http://www.diamondsearch.org/index.php, version 0.8.35). The corresponding tool HMMSCAN of the CAZy database was used to compare the non-redundant gene set with the CAZy database. The expected value of the comparison parameter was set to1e^−5^ to obtain the annotation information of the CAZymes corresponding to the gene. Then the total gene abundance of the CAZymes families was used to calculate the CAZymes abundance [17].

### Statistical analyses

Two-factor analysis of variance was used to analyze the effects of soil depth and extreme drought on different soil biochemical indexes. Repeated measures analysis of variance (ANOVA) analyzed the impacts of sampling time and extreme drought on NEE. The independent sample t test analyzed the abundance of microbial CAZymes genes affected by extreme drought in different periods, and linear fitting variance was used to test the consistency of microbial community and functional genes diversity. All tests were utilized in SPSS 22 (IBM, Armonk, USA). Pearson analysis and Mantel test were conducted using R statistical software (v.4.0.3) to identify the relationship between the CO_2_ flux and abundance of the CAZymes genes or environmental factors. A value of *P* < 0.05 was considered to be statistically significant. The contribution of microbial (bacterial and fungal) phyla to microbial CAZymes genes was done on the Majorbio Cloud Platform (www.majorbio.com).

## Results

### The effects of extreme drought on soil biochemical properties

**Fig. 1:**
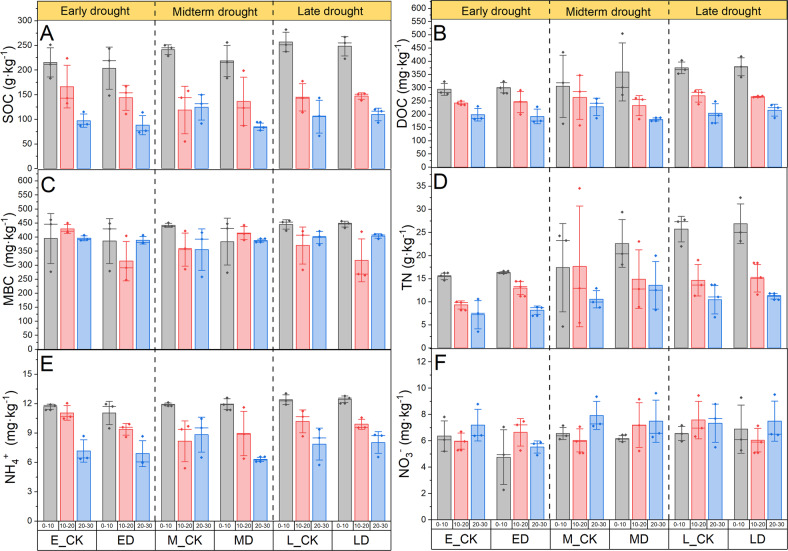
Effects of different periods of extreme drought on soil biochemical properties. **A**–**C** Changes of soil C components under extreme drought events. **D**–**F** Changes of different nitrogen components in soil under extreme drought events. Values are mean ± 1.5SE, “—” represents median line, “◆” represents outliers. SOC soil organic carbon, DOC dissolved organic carbon, MBC microbial biomass carbon, TN total nitrogen.

As shown in Fig. 1A, the range of SOC during the early, midterm and late extreme drought experiments, were 73.53–251.44 g kg^−1^, 54.75–256.16 g kg^−1^, and 66.37–282.16 g kg^−1^, respectively. Concomitantly, DOC was 171.85–323.74 mg kg^−1^, 158.15 – 504.62 mg kg^−1^, and 166.63–418.43 mg kg^−1^, MBC was 247.80 – 461.69 mg kg^−1^, 257.90–450.98 mg kg^−1^, and 264.10–458.15 mg kg^−1^, respectively (Fig. 1B, C). The variation ranges of soil TN were 3.50–16.60 g kg^−1^, 4.70–34.5 g kg^−1^, and 6.70–32.50 g kg^−1^, respectively (Fig. 1D). Similarly, the variation ranges of NH_4_^+^ were 5.96–12.03 g kg^−1^, 5.39–12.59 g kg^−1^, and 5.74–13.03 g kg^−1^, NO_3_^−^ were 2.27–8.79 mg kg^−1^, 5.07–9.62 mg kg^−1^, and 5.09–9.52 mg kg^−1^, respectively (Fig. 1E, F). The changes of SOC and NH_4_^+^ with soil depth were significantly different in different extreme drought periods and decreased significantly with the increase of soil depth (Table 1, *P* < 0.05). DOC and TN had significant differences at different depths under early and late extreme drought, which also decreased significantly with the increase in soil depth (Table 1, *P* < 0.05). The content of MBC in 10–20 cm soil was significantly lower than that in 0–10 cm soil by 22.89% (*P* < 0.05). However, soil depth had no significant effect on NO_3_^-^ in three extreme drought periods (Table 1). In this study, only the ED treatment significantly increased soil TN content by 17% (*P* < 0.05) compared with E_CK, and other soil biochemical indexes did not change significantly under extreme drought conditions (Table 1).

**Table 1:** Two-way ANOVA analysis of treatment, soil depth and their interactions on soil biochemical properties

Sources	Variables	Early drought		Midterm drought		Late drought	
		*F*	*P*	*F*	*P*	*F*	*P*
Depth	SOC	15.969	0.001	18.285	0.001	53.301	0.001
	DOC	18.518	0.001	3.260	0.074	49.049	0.001
	MBC	0.175	0.842	0.736	0.500	6.380	0.013
	TN	34.386	0.001	1.199	0.335	30.702	0.001
	NH_4_^+^	25.006	0.001	10.890	0.002	23.006	0.001
	NO_3_^−^	0.614	0.557	1.806	0.206	0.348	0.713
Treatment	SOC	0.738	0.407	0.68	0.426	0.003	0.957
	DOC	0.004	0.949	0.039	0.846	0.086	0.774
	MBC	1.944	0.189	0.144	0.711	0.433	0.523
	TN	4.923	0.047	0.193	0.669	0.204	0.659
	NH_4_^+^	2.798	0.120	0.494	0.496	0.002	0.965
	NO_3_^−^	1.752	0.210	0.035	0.855	0.220	0.648
Depth × Treatment	SOC	0.058	0.944	0.877	0.441	0.121	0.887
	DOC	0.089	0.916	0.571	0.58	0.098	0.907
	MBC	1.285	0.312	1.541	0.254	0.647	0.541
	TN	1.246	0.322	0.319	0.733	0.018	0.982
	NH_4_^+^	0.657	0.536	1.575	0.247	0.067	0.935
	NO_3_^−^	1.373	0.290	0.721	0.506	0.653	0.538

*SOC* soil organic carbon, *DOC* dissolved organic carbon, *MBC* microbial biomass carbon, *TN* total nitrogen, *NH*_*4*_^*+*^ ammonium nitrogen, *NO*_*3*_^*−*^, nitrate nitrogen.

### The effects of extreme drought on NEE

**Fig. 2:**
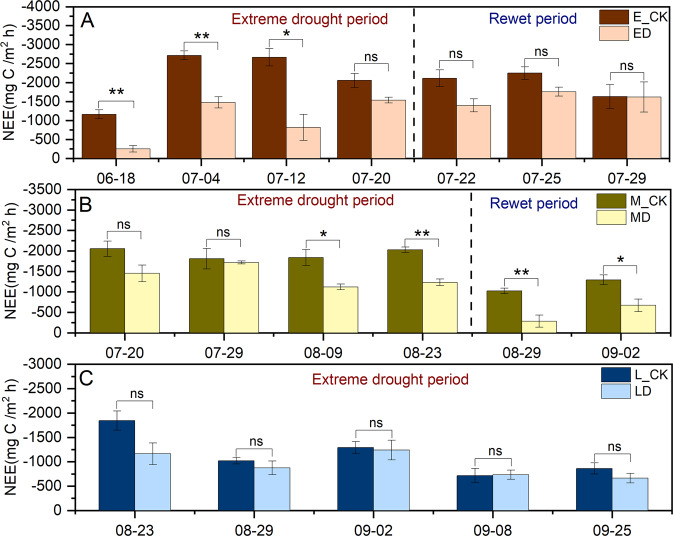
Effects of different periods of extreme drought on NEE in 2019. **A** Early extreme drought event of growing season. **B** Midterm extreme drought event of growing season. **C** Late extreme drought event of growing season. The values are shown as mean ± standard error (*n* = 3). * and ** indicate significant differences between control and extreme drought plots (Independent sample *t* test adjusted) at *P*  <  0.05 and *P*  <  0.01, ns indicates nonsignificant differences at *P*  >  0.05.

Figure 2 shows the NEE under different periods of extreme drought and Table 2 shows the difference between control and extreme drought treatments.

**Table 2:** Repeated measures ANOVA of treatment and sampling date, and their interactions on NEE in the plant growing season in 2019.

Variance source	Early drought		Midterm drought		Late drought	
	d*f*	*F*	d*f*	*F*	d*f*	*F*
Treatment (T)	1	25.88^***^	1	53.43^**^	1	7.13
Date (D)	3	63.77^***^	3	1.25	4	9.62^***^
T × D	3	13.76^***^	3	1.76	4	1.6

Significance: ***P < 0.001; **P < 0.01; *P < 0.05. NEE, net ecosystem CO_2_ exchange.

In early extreme drought, among all plots, the NEE variation range of E_CK was from −946.72 to −3093.78 mg C m^−2^ h^−1^, while the range of NEE change in ED treatment was −138.42 to −1688.88 mg C m^−2^ h^−1^ (Fig. 2A). During drought treatment, the average value of ED decreased 22–68% compared with that of E_CK. After rewetting, the gap gradually narrowed, and the range change was limited to 1–29% (Fig. 2A). The analysis of the variance of repeated measurements showed that during the drought treatment, both ED treatment and sampling time had significant effects on NEE, and the two factors showed significant interaction (Table 2, *P* < 0.05).

In midterm extreme drought, the NEE variation range of M_CK and MD were −1375.93–−2400.74 and −988.15–−1858.06 mg C m^−2^ h^−1^ respectively (Fig. 2B). During drought treatment, the average value of MD decreased 7–37% compared with that of M_CK after rewetting. The resilience of MD was found weaker than that of ED, and the carryout effect of extreme drought treatment was larger. Compared with M_CK, MD treatment decreased by 64% and 46% on the 6th and 10th day of rewetting, respectively (Fig. 2B). Even though MD had a significant effect on NEE (*P* < 0.05), the sampling time and their interaction were not significant (Table 2).

In late extreme drought, among all plots, the NEE range of L_CK varied from −464.55 to −2143.41 mg C m^−2^ h^−1^, and ranged from −496.28 to −1581.03 mg C m^−2^ h^−1^ in MD treatment (Fig. 2C). Compared with L_CK, the average value of LD per sampling decreased 4–32% during drought treatment, except 2% increase on September 8 (Fig. 2C). Compared with the previous two periods of drought, NEE was affected by LD in a lesser extent but not significantly (Fig. 2C). Even though sampling time revealed changes in NEE, time and treatment interactions did not appear significantly correlated (Table 2).

**Fig. 3:**
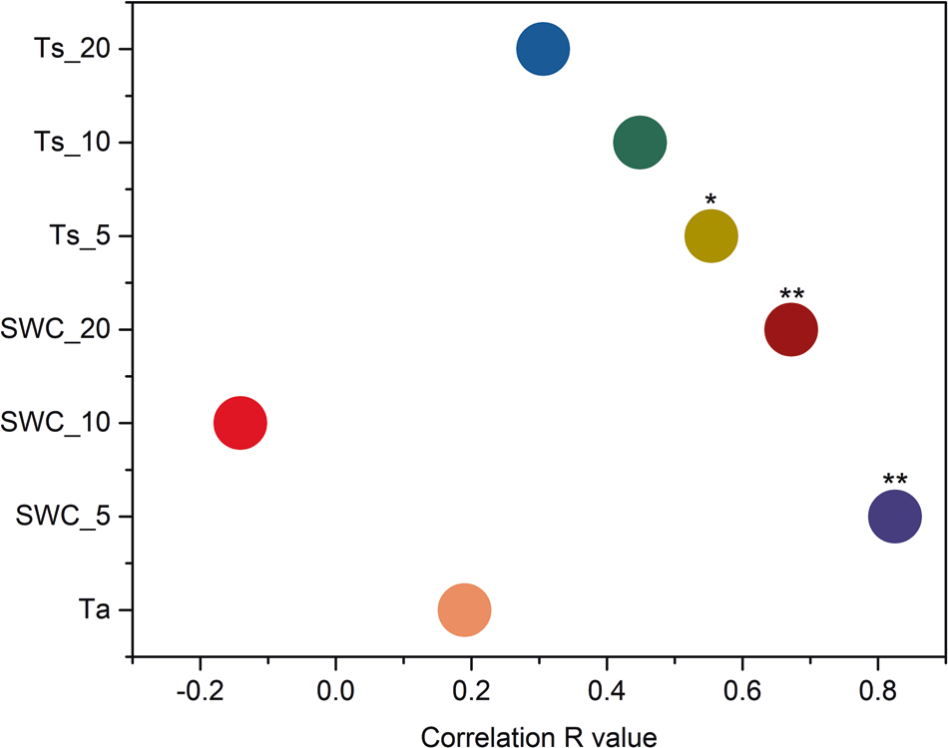
Pearson correlation coefficient between NEE and hydrothermal conditions (*n* = 108). Ta indicates air temperature. SWC_5, SWC_10 and SWC_20 indicate soil volume moisture content at 5.10 and 20 cm. Ts_5, Ts _10 and Ts_20 indicate soil temperature at 5.10 and 20 cm.

Figure 3 revealed that the change of NEE was significantly correlated with soil hydrothermal properties. SWC_5 and SWC_20 were significantly positively correlated with the change of NEE, with Pearson correlation coefficients (R) being 0.825 (*P* < 0.01) and 0.672 (*P* < 0.01), respectively. Soil temperature and NEE also showed a significant positive correlation. The correlation coefficients between Ts_5 with NEE were 0.554 (*P* < 0.05).

### Variations in CAZymes genes classes and families involved in the degradation of SOM following different periods of extreme drought

**Fig. 4:**
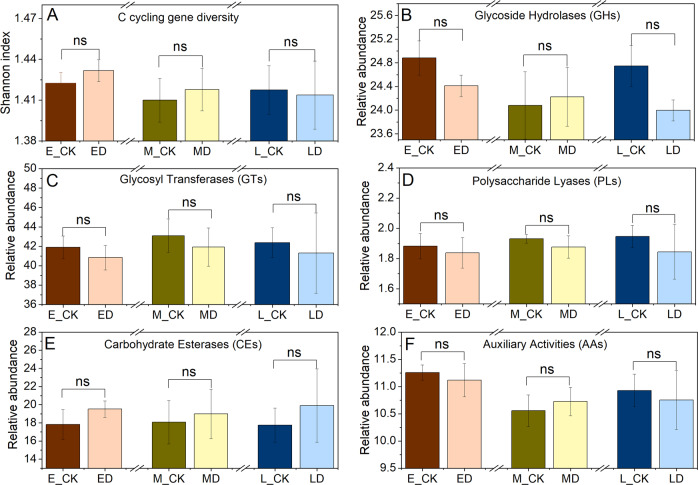
Effects of different periods of extreme drought on Shannon diversity of C cycling genes and the relative abundance of microbial CAZymes groups at class level. Differences in the **A** Shannon diversity of C cycling genes and the relative abundance of microbial gene groups encoding **B** glycoside hydrolases (GHs), **C** glycosyltransferases (GTs), **D** polysaccharide lyases (PLs), **E** carbohydrate esterases (CEs), and **F** auxiliary activities (AAs) under extreme drought at alpine peatland. The “ns” present the extreme drought effects on the relative abundance of microbial gene groups were nonsignificant at the *P* > 0.05.

Among the functional groups of CAZymes at the class level, the gene abundances of glycoside hydrolase (GHs), Glycosyl transferases (GTs), polysaccharide lyases (PLs), Carbohydrate esterases (CEs), auxiliary activities (AAs) and carbohydrate-binding modules (CBMs) are the sum of the relative abundance of each specific gene that belongs to appointed modules (Fig. 4A−F, Supplementary Fig. S1). The number of CAZymes families in GHs, GTs, PLs, CEs, AAs and CBMs were 111, 69, 18, 16, 10 and 40, respectively. Although GHs has a great advantage in quantity, GTs relative abundance was the largest, ranging from 38.40% to 40.04% (Fig. 4C), while CBMs varied only from 2.09% to 2.15% (Supplementary Fig. S1) for all the plots. At the family level, GT41 (peptide beta-N-acetylglucosaminyltransferase: EC 2.4.1.225), GT4 (sucrose synthase: EC 2.4.1.13), GT2 (cellulose synthase: EC 2.4.1.12), CE1 (acetyl xylan esterase: EC 3.1.1.72) and CE10 (arylesterase: EC 3.1.1.2) presented the top five highest relative abundance in both control and extreme drought treatments.

**Fig. 5:**
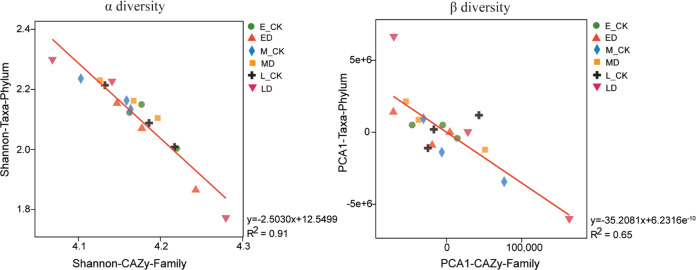
Correlations between soil microbial community and CAZyme families. Species and functional regression analysis for α and β diversity between CAZyme families and soil microbial community at phylum level (*P* < 0.001) of control and extreme drought plots at alpine peatland.

In all samples of this study, taxon statistics and distribution for GHs, GTs, PLs, CEs, AAs, and CBMs were shown in Supplementary Table S1. In general, the relative abundance of microorganisms for GHs and GTs at phylum, class, order, family, genus, and species levels were higher than other CAZymes families. Figure 5 showed that the regression between microbial species and functional genes was significantly correlated (*P* < 0.001) for α and β diversity under extreme drought in the alpine peatland.

We selected specific CAZyme families which are involved in the decomposition of SOM, including plant-derived biomass, such as starch, hemicellulose, carbohydrate esters, pectin, and lignin, fungal-derived biomass such as chitin and glucan, and bacteria-derived biomass such as peptidoglycan. In general, the recalcitrance of organic C components derived from plants follows the order: starch, which is the most easily mineralizable, hemicellulose, carbohydrate esters, pectin, and lignin, which is the most resistant. Enzymatic depolymerization of the different soil organic C components requires the synergistic action of a spectrum of enzymes as shown in Supplementary Table S2. Enzymes contributing to plant-derived biomass decomposition are classified into GHs, CEs, AAs, and PLs families. Enzymes contributing to both the fungal- and bacteria-derived biomass are classified into the GHs families.

**Fig. 6:**
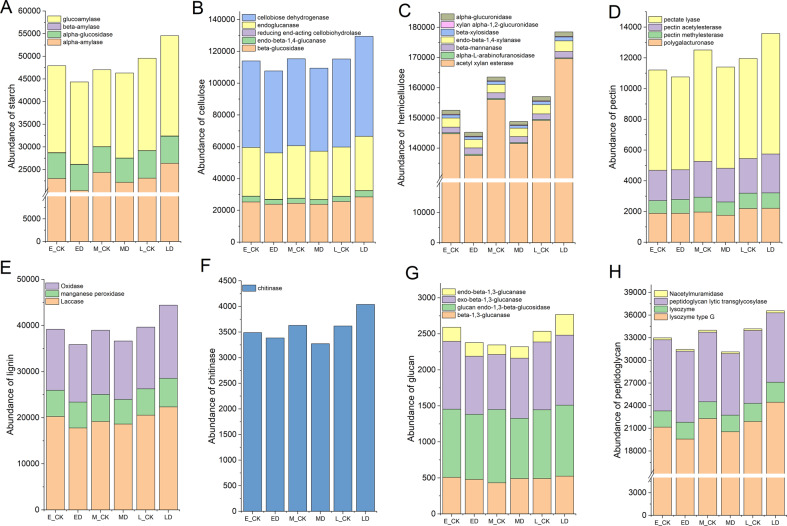
The abundance of selected CAZymes involved in the decomposition of the SOM following different periods of extreme drought. **A**–**E** indicate the enzymes mainly involved in the starch, cellulose, hemicellulose, pectin and lignin decomposition, which were organic matters derived from plants. **F**, **G** indicate the enzymes mainly involved in the chitinase and glucan decomposition, which were organic matters derived from fungus. **H** indicate the enzymes mainly involved in the peptidoglycan decomposition that are derived from bacteria.

As shown in Fig. 6, for the decomposition of different SOM, the CAZymes families involved in the enzymes had the same variation trend. They decreased under ED and MD, while increased under LD compared to control. For the decomposition of starch, the alpha-amylase and glucoamylase had a higher abundance of specific microbial CAZymes families compared to alpha-glucosidase and beta-amylase (Fig. 6A). The cellobiose dehydrogenase, acetyl xylan esterase and pectate lyase were the microbial CAZymes families with the highest abundance of cellulose, hemicellulose and pectin (Fig. 6B, C, D). The three main enzymes that participated in the decomposition of lignin were oxidase, manganese peroxidase, and laccase, with manganese peroxidase being the least abundant among them (Fig. 6E). For the decomposition of fungal-derived biomass such as chitin and glucan, chitinase was the most abundant, while endo-1,3-glucanase was the least abundant (Fig. 6F, G). From the four main enzymes involved in the bacteria-derived biomass, the higher gene number of microbial CAZymes families was related to lysozyme type G and peptidoglycan lytic transglycosylase (Fig. 6H).

**Fig. 7:**
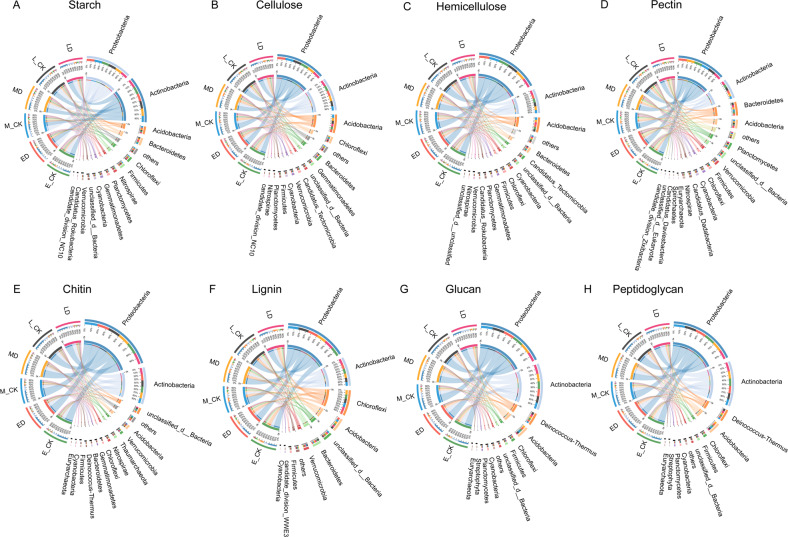
Contribution of microbial (bacterial and fungal) phyla to microbial CAZymes genes for SOM decomposition following different periods of extreme drought. **A**–**E** indicate the microbial community involved in the starch, cellulose, hemicellulose, pectin and lignin decomposition, which were organic matters derived from plants. **F**, **G** indicate the microbial community involved in the chitinase and glucan decomposition, which were organic matters derived from fungus. **H** indicate the microbial community mainly involved in the peptidoglycan decomposition that derived from bacteria.

Family level CAZymes genes involved in the selected different types of SOM decomposition were attributed to the bacterial community, mainly to *Proteobacteria* and *Actinobacteria* (Fig. 7). In addition to the two bacterial communities above, the other microbial communities contributed to different types of SOM decomposition genes existed differences as expected. For starch, hemicellulose, and pectin, *Acidobacteria* and *Bacteroidetes* were the main species (Fig. 7A, C, D). For cellulose and chitin, the main species contributing were *Chloroflexi* and *Acidobacteria* (Fig. 7B, E). Besides the top two communities that contributed to the genes, unclassified bacteria were also present in lignin decomposition (Fig. 7F). *Deinococcus-Thermus* and *Acidobacteria* control glucan and peptidoglycan decomposition (Fig. 7G, H).

### The role of microbial CAZymes genes of SOM degradation in characterizing ecosystem CO_2_ fluxes

**Fig. 8:**
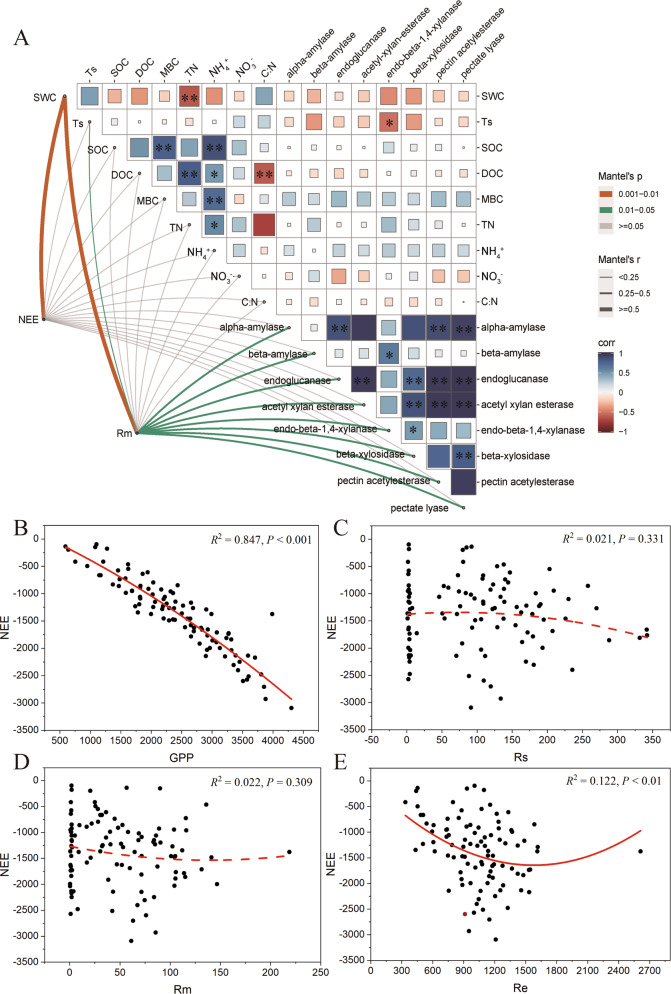
Relationships among CO_2_ fluxes, environmental factors, and CAZymes genes encoding SOM decomposition enzymes. **A** Mantel test between the abundance of CAZymes genes encoding SOM decomposition enzymes and environmental factors. Mantel’s *r* and *P* values are indicated based on the color and the width of the connecting lines as specified in the figure legend. SWC, soil volumetric water content, Ts soil temperature, SOC soil organic carbon, DOC dissolved organic carbon, MBC microbial biomass carbon, TN total nitrogen. **B**–**E** The fitting curve of NEE and other carbon fluxes. NEE net ecosystem CO_2_ exchange, GPP gross primary productivity, Rs soil respiration, Rm microbial respiration, Re ecosystem respiration.

We found that ED and MD significantly decreased NEE (Table 2, *P* < 0.05), while LD significantly decreased the Rm [18]. Moreover, Rm greatly varied with the specific CAZymes involved in the decomposition of starch, cellulose, hemicellulose and pectin, while NEE was only affected by soil hydro-thermal Factors (Fig. 3, Fig. 8A). In detail, Rm was positively and significantly correlated with alpha-amylase (*R* = 0.31, *P* < 0.05), beta-amylase (*R* = 0.26, *P* < 0.05), endoglucanase (*R* = 0.43, *P* < 0.05), acetyl xylan esterase (*R* = 0.37, *P* < 0.05), endo beta-1.4-xylanase (*R* = 0.41, *P* < 0.05), beta-xylosidase (*R* = 0.37, *P* < 0.05), pectin-acetylesterase (*R* = 0.38, *P* < 0.05) and pectate-lyase (*R* = 0.43, *P* < 0.05). Both NEE and Rm were negatively and significantly correlated with the soil water content. Surprisingly, soil C and N components were not significantly correlated with the CAZymes (Fig. 8A).

We assessed the correlation between NEE and GPP, Rs, Rm, and Re by the polynomial Fit. GPP is not only one of the major determinants of carbon exchange between the atmosphere and terrestrial ecosystems, but also a crucial gauge to describe plant activities and functions.a strong and significant correlation was found between NEE and GPP (*n* = 108, *R*^2^ = 0.847, *P*  <  0.001, Fig. 8B). A weak but significant correlation was found between NEE and Re (*n* = 108, *R*^2^ = 0.122, *P*  <  0.01, Fig. 8C). For CO_2_ fluxes from soils, both the Rs and Rm were nonsignificantly correlated with NEE (Rs: *n* = 108, *R*^2^ = 0.021, *P* = 0.331, and Rm: *n* = 108, *R*^2^ = 0.022, *P* = 0.309, Fig. 8D, E).

## Discussion

### Net CO_2_ sink function decreased under extreme drought in alpine peatland

Studies have shown that the intensity of CO_2_ sinks is greater in the northern biota, with observed and predicted annual mean NEE of −46 g C m^−2^ yr^−1^ and −29 g C m^−2^ yr^−1^, respectively, and +10 g C m^−2^ yr^−1^ and −2 g C m^−2^ yr^−1^ in the tundra, respectively [30]. In our study, the variation range of NEE in control plots during the growing season was higher than the annual mean value of the northern biota (Fig. 2). Although the ED and MD significantly reduced the net CO_2_ absorption capacity of Zoige peatland, the overall C sink function remained unchanged. However, due to the short net CO_2_ uptake period (73 ± 7 days) and high respiration, the Peatlands of the Italian Alps remain a C source based on CO_2_ emissions (NEE: 180.7 ± 65.2 g C single bond CO_2_ m^−2^ yr^−1^) under high interannual weather conditions for three consecutive years [31]. After an extreme drought event and rewetting, recovery in ED is faster than in MD. It can be concluded that different periods of extreme drought events had different effects on the CO_2_ sink function of Zoige peatland. Specifically, extreme drought events that occurred in the early and midterm of the growing season lead to significantly positive C-climate feedback by reducing CO_2_ absorption of the ecosystem. This is consistent with previous studies for the viewpoint that climate variability occurring in the early or midterm stage of the growing season could have larger impacts than late in the growing season [12, 13].

The change of soil moisture drives 90% of the interannual variation of global land C absorption, mainly through its influence on photosynthesis [32]. In Sphagnum-dominated peatland, there is a linear relationship between soil bulk water content and NEE. Moist sites act as net CO_2_ sinks (up to −5 g CO_2_ m^−2^ day^−1^), while sites closest to ditches (simulated extreme drought) are always small C sources [33]. Our study also pointed out that soil water content and temperature had a significantly positive correlation with NEE. Therefore, the reduction of soil water content caused by extreme drought is an important driving factor for the reduction of C absorption in the peatland ecosystem.

### The decomposition potential of microorganisms to SOM shows resilience following disturbances from extreme drought

GHs and GTs are two major classes of enzymes known for catalytic hydrolysis and/or synthesis of glycosidic bonds between carbohydrates [34]. In our study, the GHs and GTs families were the most abundant CAZymes among the treatments, which is similar to many metagenomic soil studies [35], and may be attributed to the fact that GHs and GTs are more abundantly available in genomic databases, currently numbering 173 and 115 families respectively, as curated on the CAZymes database. On the other hand, PLs, CEs and AAs have just 42, 20, and 17 families known to date [17, 18, 36].

Our results indicated that for the decomposition of different plant-derived biomass, α-amylase (EC 3.2.1.1), cellobiose dehydrogenase (EC 1.1.99.18), acetyl xylan esterase (EC 3.1.1.72), pectate lyase (EC 4.2.2.2) and manganese peroxidase (EC 1.11.1.13) have the highest abundance of microbial CAZymes genes families for the decomposition of starch, cellulose, hemicellulose, pectin and lignin respectively. Various organisms, such as plants, animals, bacteria, fungi and yeast, secrete α-amylase (EC 3.2.1.1) as an extracellular enzyme, which is an important hydrolase widely distributed in soil [37–39]. Cellobiose dehydrogenase (EC 1.1.99.18), isolated from a cellulose-degrading culture of *P. chrysosporium*, is a fungal flavoenzyme used as a bioelectrocatalyst and is capable of inducing direct and mediated electron transfer [40, 41]. Hemicellulose is the second largest cell wall polysaccharide interwoven between cellulose and lignin. In our study, acetyl xylan esterase (EC 3.1.1.72) genes have the highest abundance, which is responsible for hydrolyzing acetyl substitutions on xylan side chains [42, 43]. Pectate lyases (EC 4.2.2.2) is secreted by bacterial and fungal pathogens and catalyze the degradation of pectin acid components in intermediate lamella and cell walls of higher plants [44]. The extracellular enzyme manganese peroxidase (EC 1.11.1.13) is believed to degrade lignin through the oxidation of hydrogen peroxide-dependent Mn (II) to Mn (III) [45]. Chitin is composed of large, crystalline N-containing polysaccharides formed by N-acetylglucosamine chains. It is even considered to be one of the most abundant biomolecules on earth, with an estimated annual production of 10^11^–10^14^ tons [46]. Peptidoglycan consists of short peptide crosslinked alternating β-1, 4-acetylglucosamine and N-acetyltetracylinic acid units [47]. For the decomposition of fungal-derived biomass, chitinase (EC 3.2.1.14) and glucan endo-1, 3-beta-glucosidase (EC 3.2.1.39) genes had the most abundant, and lysozyme type G (EC 3.2.1.17) has the most abundance for the peptidoglycan, which derives from bacterial biomass.

For the decomposition of different SOM components, the microbial CAZyme genes were not significantly affected by extreme drought treatments, which indicated the resilience of microbial function to extreme drought. Similarly, many studies have reported on the resistance and resilience of soil microbial communities to extreme weather events, as they are frequently exposed to seasonal drought and are able to recover after rewetting [48–50]. Although insufficient rainfall leads to a decrease of microbial biomass and activity, the microbial response to experimental drought is weak [51].

In our study, CAZymes genes in all selected soil organic matter were largely derived from the bacterial community, mainly *Proteobacteria* and *Actinobacteria* (Fig. 7). A previous study has also shown that a high frequency of genes observed in bacterial genomes is involved in the degradation of structural plant polysaccharides [52]. For different types of SOM, the communities that contribute to degradation were also different. It has been reported that the abundance of *Acidobacteria* is negatively correlated with the mineralization rate of C, while the abundance of *Proteobacteria* and *Bacteroidetes* is positively correlated with C mineralization rates [53]. It has also been pointed out that the response of microorganisms to experimental drought is weak, the scant rainfall period leads to the decrease of microbial biomass and activity, and the relative abundance of bacterial community [48]. Therefore, by distinguishing the various species’ functions and their contribution to SOM, we can predict the ecological properties of various bacterial taxa to better understand the structure and function of soil microbial communities.

### Microbial enzymatic degradation of SOM exerts a weaker role than plants in influencing NEE under extreme drought

The current evidence showed that NEE was mainly affected by soil hydrothermal properties including SWC and soil temperature. Besides that, the strong correlation between GPP and NEE demonstrated the critical role of plants, while microbial enzymatic decomposition genes were weak indicators of NEE, which was due to their asynchronous responses to extreme drought events. Although research has shown that microbial community is very sensitive to precipitation changes, even slight precipitation events can cause rapid microorganisms’ responses [54]. While mounting evidence suggests that changes in community structure can occur as adaptive responses to drying-rewetting cycles [51, 55], we proved the relative stability of microbial SOM decomposition function from the perspective of metagenomics. One possible explanation was that belowground-based studies have reported the resistance and resilience of soil microbial communities to extreme weather events due to their routine exposure to seasonal drought and ability to recover upon rewetting [49, 56–59].

Rm is a major process that controls C release from terrestrial ecosystems to the atmosphere [60]. Rm rates are primarily driven by substrate availability, temperature, and moisture, roughly 30% of observed variation cannot be explained by these factors [61]. The earlier observations indicated that the temperature sensitivity of Rm varied with the interactive effect between soil moisture and sampling location [62]. Some previous experiments have also revealed that Rm regulation by soil moisture was associated with shifts in enzymatic activities and carbon use efficiency [63, 64], and suggested that future drought might weaken the thermal compensatory capacity of Rm, with important consequences for C-climate feedback [65]. Our study furtherly revealed that Rm had a significant positive correlation with specific microbial CAZymes genes involved in starch, cellulose, hemicellulose, and pectin, that belonged to labile C of soil. In our previous study, we also found that SWC, available phosphorus, and aromatic compound degraders were also the main driving forces of Rm (*P* < 0.05) [23].

Under the dual C goal project (C neutralization and C peak) in China, peatland is an important ecosystem of the C repository. By the control of microbial communities and C inputs, peatlands can alter their C sink functions to counteract the fallout from climate change. It has been showed that hydrological conditions were the primary determinant of interannual C fluxes variations on the Zoige peatlands [66], under drought condition, aerobic decomposition accelerates and peatlands release CO_2_. Conversely, decomposition slows and peat accumulates in an anaerobic environment [67, 68]. Our results indicated the relative stability of microbial SOM decomposition genes from the perspective of metagenomics under repeated extreme drought events, indicating that although the CO_2_ sink function of peatland ecosystem significantly reduced, the soil C pool seems to be more stable than expected. Overall, even though DNA- and RNA-based meta-genomic techniques have provided insight into the microbiome compositions of soils, they may not relate well to ecosystem processes or functions. The relationship between the abundances of rRNA and mRNA of CO_2_-cycling microbes needs further study [69].

## Conclusions

Our research pointed out that early and midterm extreme drought events of the growing season reduced the CO_2_ sink capacity of Zoige peatland significantly, which is mainly driven by hydrothermal factors and gross primary productivity from plants but weakly associated with soil organic matter degradation genes from the perspective of CAZymes profiling. Besides, soil microbial respiration was significantly and positively related to specific key CAZymes genes involved in labile carbon degradation processes. Because of the great influence of plants, it is verified that microbial enzymatic genes still have a certain lag in the characterization of net ecosystem CO_2_ exchange, yet how to add microbial processes to the ecosystem model for explicit expression and parameterization, and which year, after repeated extreme drought events occurred, will significantly increase the decomposition of soil organic matter and bring huge threat to soil carbon pool of alpine peatland, need further study, which could help the development of strategies for stabilizing and increasing peatland carbon stocks.

## Supplementary information

Supplementary information

## Data Availability

The datasets generated and/or analysed during the current study are available from the corresponding author on reasonable request. Sequence data associated with this project have been deposited in the National Center for Biotechnology Information (NCBI) under the accession number SRP368675.
