# Supplementary material for: Asynchronous responses of microbial CAZymes genes and the net CO2 exchange in alpine peatland following 5 years of continuous extreme drought events
Source: ISME Commun. 2022 Nov 16;2:115. doi: 10.1038/s43705-022-00200-w (PMC9723601; doi:10.1038/s43705-022-00200-w)
Supplement: Supplementary file 1 — Supplementary information [file 43705_2022_200_MOESM1_ESM.docx]

**Asynchronous responses of microbial CAZymes genes and the net CO_2_ exchange in alpine peatland following 5 years of continuous extreme drought events**

Zhongqing Yan^1,2,3^, Enze Kang^1,2,3^, Kerou Zhang^1,2,3^, Yanbin Hao^4^, Xiaodong Wang^1,2,3^, Yong Li^1,2,3^, Meng Li^1,2,3^, Haidong Wu^5^, Xiaodong Zhang^1,2,3^, Liang Yan^1,2,3^, Wantong Zhang^6^, Jie Li^7^, Ao Yang^1,2,3^, Yuechuan Niu^4^, Xiaoming Kang^1,2,3^*

^1^ Wetland Research Center, Institute of Ecological Conservation and Restoration, Chinese Academy of Forestry, Beijing 100091, China;

^2^ Sichuan Zoige Wetland Ecosystem Research Station, Tibetan Autonomous Prefecture of Aba 624500, China;

^3^ Beijing Key Laboratory of Wetland Services and Restoration, Beijing 100091, China;

^4^ College of Life Sciences, University of Chinese Academy of Sciences, Beijing 100049, China;

^5^ Information Center of Ministry of Ecology and Environment, Beijing 100029, China;

^6^ Sino-Danish Centre for Education and Research, University of Chinese Academy of Sciences, Beijing 100049, China

^7^ State Key Laboratory of Mycology, Institute of Microbiology, Chinese Academy of Sciences. Beijing 100101, China

***Corresponding author：**

Prof. Xiaoming Kang, Wetland Research Center, Institute of Ecological Conservation and Restoration, Chinese Academy of Forestry, Beijing 100091, China, contactable by email address xmkang@ucas.ac.cn. The primary work telephone number is +86 (010) 6282 4153.

**Keywords:** Alpine peatland, Extreme drought, NEE, Metagenomics, Microbial CAZymes families

**Supplementary Figures**


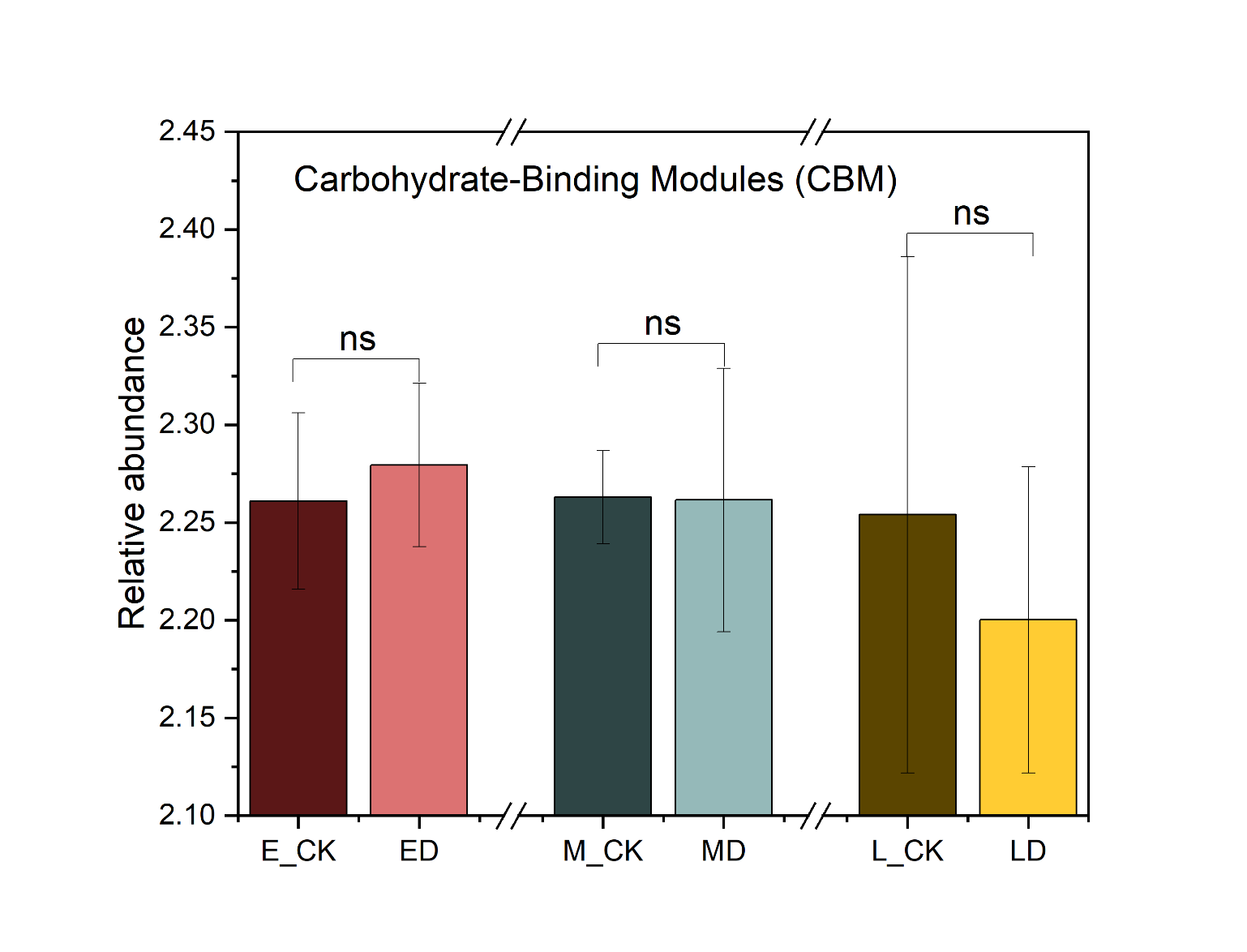


# **Fig. S1.** Differences in the relative abundance of microbial gene groups encoding carbohydrate-binding modules (CBMs) under extreme drought at alpine peatland. The values are shown as mean ± standard error (n = 3). ns indicates nonsignificant differences at *P* > 0.05.

**Table S1.** Taxon statistics for different CAZyme families.

| CAZyme family | Domain | Kingdom | Phylum | Class | Order | Family | Genus | Species |
| --- | --- | --- | --- | --- | --- | --- | --- | --- |
| GHs | 5 | 8 | 101 | 181 | 326 | 532 | 1554 | 5678 |
| GTs | 5 | 8 | 110 | 203 | 391 | 646 | 1796 | 6614 |
| PLs | 5 | 8 | 63 | 115 | 202 | 327 | 794 | 1780 |
| CEs | 4 | 7 | 87 | 158 | 289 | 482 | 1419 | 4851 |
| AAs | 5 | 8 | 70 | 137 | 263 | 432 | 1173 | 3795 |
| CBMs | 5 | 8 | 62 | 114 | 201 | 335 | 924 | 2206 |

**Table S2.** The detailed information of CAZymes families involved in the degradation of starch, cellulose, hemicellulose, pectin, chitin and lignin.

| Organic components | Enzymes | CAZymes families |
| --- | --- | --- |
| Starch | alpha-amylase (EC 3.2.1.1) | GH13, GH119, GH57 |
|  | alpha-glucosidase (EC 3.2.1.20) | GH31 |
|  | beta-amylase (EC 3.2.1.2) | GH14 |
|  | glucoamylase (EC 3.2.1.3) | GH15, GH97 |
| Cellulose | beta-glucosidase (EC 3.2.1.21) | GH1, GH3, GH116 |
|  | endo-beta-1,4-glucanase (EC 3.2.1.4) | GH5 |
|  | reducing end-acting cellobiohydrolase (EC 3.2.1.176) | GH48 |
|  | endoglucanase (EC 3.2.1.4) | GH124, GH45, GH6, GH9, GH51, GH74, GH12, GH44 |
|  | cellobiose dehydrogenase (EC 1.1.99.18) | AA3 |
| Hemicellulose | acetyl xylan esterase (EC 3.1.1.72) | CE1, CE2, CE3, CE4, CE5, CE6, CE7 |
|  | alpha-L-arabinofuranosidase (EC 3.2.1.55) | GH62, GH54 |
|  | beta-mannanase (EC 3.2.1.78) | GH113, GH26 |
|  | endo-beta-1,4-xylanase (EC 3.2.1.8) | GH10, GH11, GH30 |
|  | beta-xylosidase (EC 3.2.1.37) | GH120, GH43, GH52 |
|  | xylan alpha-1,2-glucuronidase (3.2.1.131) | GH115 |
|  | alpha-glucuronidase (EC 3.2.1.139) | GH67 |
| Pectin | polygalacturonase (EC 3.2.1.15) | GH28 |
|  | pectin methylesterase (EC 3.1.1.11) | CE8 |
|  | pectin acetylesterase (EC 3.1.1.-) | CE12, CE13 |
|  | pectate lyase (EC 4.2.2.2) | PL1, PL2, PL3, PL9, PL10 |
| Lignin | Laccase (EC 1.10.3.2) | AA1 |
|  | manganese peroxidase (EC 1.11.1.13) | AA2 |
|  | Oxidase (EC 1.1.3.-) | AA5 |
| Chitin | chitinase (EC 3.2.1.14) | GH18, GH19 |
| Glucan | beta-1,3-glucanase (EC 3.2.1.39) | GH64, GH128 |
|  | glucan endo-1,3-beta-glucosidase (EC 3.2.1.39) | GH17 |
|  | exo-beta-1,3-glucanase (EC 3.2.1.58) | GH55 |
|  | endo-beta-1,3-glucanase (EC 3.2.1.39) | GH81 |
| peptidoglycan | lysozyme type G (EC 3.2.1.17) | GH23 |
|  | lysozyme (EC 3.2.1.17) | GH24, GH25, GH73 |
|  | peptidoglycan lytic transglycosylase (EC 3.2.1.-) | GH102, GH103, GH104 |
|  | N-acetylmuramidase (EC 3.2.1.17) | GH108 |
